# Allelic Interaction between *CRELD1* and *VEGFA* in the Pathogenesis of Cardiac Atrioventricular Septal Defects

**DOI:** 10.3934/genet.2014.1.1#sthash.jksuJTeC.dpuf

**Published:** 2014

**Authors:** Jennifer K. Redig, Gameil T. Fouad, Darcie Babcock, Benjamin Reshey, Eleanor Feingold, Roger H. Reeves, Cheryl L. Maslen

**Affiliations:** 1 Department of Molecular and Medical Genetics, Oregon Health & Science University, Portland, OR 97239, USA; 4 Department of Biomedical Engineering, Oregon Health & Science University, Portland, OR 97239, USA; 5 Knight Cardiovascular Institute, Oregon Health & Science University, Portland, OR 97239, USA; 6 Department of Human Genetics, Graduate School of Public Health, University of Pittsburgh, Pittsburgh PA 15261, USA; 7 Department of Physiology and the Institute for Genetic Medicine, Johns Hopkins University School of Medicine, Baltimore, MD 21205, USA

**Keywords:** congenital heart disease, genetic modifier

## Abstract

Atrioventricular septal defects (AVSD) are highly heritable, clinically significant congenital heart malformations. Genetic and environmental modifiers of risk are thought to work in unknown combinations to cause AVSD. Approximately 5–10% of simplex AVSD cases carry a missense mutation in *CRELD1*. However, *CRELD1* mutations are not fully penetrant and require interactions with other risk factors to result in AVSD. Vascular endothelial growth factor-A (VEGFA) is a well-characterized modulator of heart valve development. A functional *VEGFA* polymorphism, *VEGFA* c.–634C, which causes constitutively increased VEGFA expression, has been associated with cardiac septal defects suggesting it may be a genetic risk factor. To determine if there is an allelic association with AVSD we genotyped the *VEGFA* c.–634 SNP in a simplex AVSD study cohort. Over-representation of the c.–634C allele in the AVSD group suggested that this genotype may increase risk. Correlation of *CRELD1* and *VEGFA* genotypes revealed that potentially pathogenic missense mutations in *CRELD1* were always accompanied by the *VEGFA* c.–634C allele in individuals with AVSD suggesting a potentially pathogenic allelic interaction. We used a *Creld1* knockout mouse model to determine the effect of deficiency of Creld1 combined with increased VEGFA on atrioventricular canal development. Morphogenic response to VEGFA was abnormal in Creld1-deficient embryonic hearts, indicating that interaction between CRELD1 and VEGFA has the potential to alter atrioventricular canal morphogenesis. This supports our hypothesis that an additive effect between missense mutations in *CRELD1* and a functional SNP in *VEGFA* contributes to the pathogenesis of AVSD.

## 1. Introduction

Congenital heart defects (CHD) are the most common form of birth defect, with an incidence of nearly 9 per 1,000 live births [1]. The genetic basis remains largely elusive even though the evidence for genetic contributions to CHD is pervasive [2]. Since most CHD occurs sporadically, it is clear that genetic modifiers contribute to risk, but the evidence for contributions from specific genes is lacking. To date few studies have identified genetic risk factors for CHD or, more importantly, interactions between modifiers that further increase risk and cause disease. To do so requires complimentary studies in human genetics and animal models to first find the genes at play in causing human heart malformations and then demonstrate that allelic interactions between risk factors actually occur *in vivo* to deleteriously modify heart development. Here we explore a potential allelic interaction between two CHD risk factors and the implications for the pathogenesis of CHD in humans.

Atrioventricular septal defect (AVSD) is one of the most severe cardiac septal defects, occurring in 2.4–3.1/10,000 live births. AVSD results from incomplete septation of the atrioventricular (AV) canal, including defective AV valve formation. This incomplete septation allows oxygenated and un-oxygenated blood to mix, increases the heart’s workload, and untreated can cause congestive heart failure and death. Surgical repair has greatly increased survival and quality of life, but the repaired AVSD population remains at higher risk for morbidity and mortality [3,4]. Understanding the genetic contributions to AVSD has profound implications for the long-term management of this defect and risk to future generations.

Approximately 65% of all AVSD occurs in individuals with Down syndrome (DS), demonstrating a contribution of large effect by trisomy 21. However, about 50% of Down syndrome children are born with a structurally normal heart, indicating that additional risk factors are required to manifest the defect even on this highly sensitized genetic background. The identification of missense mutations in VEGFA pathway genes in our recent study of Down syndrome-associated AVSD suggests that genetic variants not on chromosome 21 contribute to the etiology of AVSD in the context of trisomy 21 [5]. Inheritance of these mutations from an unaffected parent demonstrates that these incompletely penetrant alleles exist in the general population as benign variants that become pathogenic on a susceptible genetic background. Consequently, they are likely to contribute to the underpinnings of AVSD in the euploid population as well. This has been demonstrated for the *AVSD2* gene (MIM 607170), *CRELD1*, where missense mutations occur in both simplex AVSD and Down syndrome-associated AVSD [6,7]. One recurrent *CRELD1* missense mutation, p.Arg329Cys, has been shown to cause protein misfolding [7], and others are predicted to be inactivating as well [5]. We confirmed the status of *CRELD1* mutations as modifiers for heart defects using a mouse model, where loss-of-function for CRELD1 was shown to increase cardiac septal defects when expressed on a Down syndrome mouse model background [8]. *Creld1*^+/−^ mice were crossed with the Ts65Dn strain, a widely used model for DS that is trisomic for approximately half of the orthologs on human chromosome 21. Reduced expression of CRELD1 resulted in a significant increase in cardiac septal defects in the offspring demonstrating that loss-of-function for CRELD1 is a genetic modifier for CHD. However, *CRELD1*-missense mutations are incompletely penetrant on a euploid background [6,7] and the additional modifiers required to cause simplex AVSD are unknown.

AVSD has traditionally been called an endocardial cushion defect as it was once presumed to arise from improper development of the AV endocardial cushions, which are anlagen of the AV valves and septa [9]. However, we now know that during AV septation a second heart field-derived structure called the dorsal mesenchymal protrusion (DMP) and the mesenchymal cap of the primary atrial septum fuse with the AV endocardial cushions to form a complex that eventually develops into the mature AV valves in the properly septated four-chambered heart [10]. Consequently the developmental basis for AVSD is not necessarily due solely to endocardial cushion defects, although the AV cushions do play important role in that process. Here we explore a potential role for the AV cushions in AVSD mediated by mutations in the AVSD risk gene, *CRELD1*.

VEGFA is a potent mitogen known to modulate endocardial cushion development [11,12]. The AV endocardial cushions are the source of a population of cells that arise through an epithelial to mesenchymal transformation during AV canal morphogenesis and contribute to formation of the AV valves and septa. VEGFA signaling directs the morphogenesis of the AV endocardial cushions into the mature valve, with expression levels under tight control throughout this process [13]. However, individual differences in constitutive VEGFA expression exist and have been the source of much speculation about VEGFA expression levels and susceptibility to a multitude of disease phenotypes. Differential regulation of VEGFA expression is in part under the control of a functional single nucleotide polymorphism (SNP) in the 5′ UTR of *VEGFA* at position c.–634 (rs2010963) [14,15]. The c.–634C/G SNP alters a ribosome entry site affecting translation of VEGFA. The c.–634C allele has been shown to result in increased post-transcriptional VEGFA expression compared to expression from the c.–634G allele. The up-regulating c.–634C allele has been associated with a heterogeneous group of cardiac septal defects in case-control studies [16,17]. Consequently, it appears that the c.–634C SNP may be a genetic risk factor for cardiac septal defects, presumably as a result of increased expression of VEGFA. In a hypermorphic mouse model, a modest increase in VEGFA expression of two to three-fold resulted in severe abnormalities in heart development and early embryonic death [18]. However, with a minor allele frequency of 0.358, the human *VEGFA* c.–634C variant appears to be harbored in the general population as a generally benign allele, but with the potential to contribute to disease pathogenesis when expressed on an otherwise genetically sensitized background.

To date, the identification of genetic risk factors for AVSD in humans has largely occurred through the characterization of individual variants found to be associated with the disease phenotype. Yet we know that AVSD is a complex trait that requires a yet unknown combination of factors to breach the theoretical disease threshold [5,8]. Consequently, it is important to identify biological interactions between protein variants associated with AVSD to identify those with additive or synergistic effects that may confer disease. Here we investigate the co-segregation of *CRELD1* missense mutations and the *VEGFA* c.–634C allele in individuals with simplex AVSD, and characterize an interaction between CRELD1 and VEGFA in the developing heart as a possible contributing mechanism in the pathogenesis of this clinically significant heart malformation.

## 2. Subjects and Methods

### 2.1. Study Participants

Study subjects were recruited through the Oregon Congenital Heart Disease Registry (ORCHD), a population-based registry of all Oregon patients born with one of 14 major heart defects and who have heart surgery before the age of 19. ORCHD categorizes the registry based on clinical diagnosis of the heart defect from surgical records insuring homogeneity within phenotypic categories. All study subjects were recruited under a protocol approved by the institutional review board with informed consent by the subject or custodial parent. Inclusion criteria for this study were based on the diagnosis of non-syndromic isolated AVSD, including complete AVSD and partial AVSD (also known as ostium primum atrial septal defect). There was no evidence of any extra-cardiac anomalies in this cohort. In total we recruited 29 individuals with a complete AVSD and 21 individuals with a partial AVSD. All study subjects were self-reported non-Hispanic white. DNA specimens were obtained from all study subjects by either a peripheral blood draw or saliva collection. One hundred race matched controls were obtained from the Coriell Caucasian Human Variation Control DNA panel (HD100CAU, Coriell Institute for Medical Research, Camden NJ). We did not control for age or gender.

### 2.2. VEGFA Genotyping

Genomic DNA was extracted from blood or saliva samples from study participants using standard techniques. Control DNA was from the Coriell Caucasian Human Variation Control panel. The *VEGFA* c.–634 genotype was determined by Sanger sequencing of genomic DNA-derived PCR amplicons encompassing *VEGFA* c.–634 as the template. All samples for cases and controls were resequenced by the core sequencing laboratory at the Oregon Clinical Translational Research Institute (OCTRI) at OHSU. Electropherogram traces were transferred to the Maslen lab electronically and the traces were interpreted using MutationSurveyor software.

### 2.3. Creation of the Creld1-knockout mouse

To remove *Creld1*from the mouse genome we used a modified pACN-1 vector (a gift from Dr. Kirk Thomas, University of Utah). The completed knockout cassette (Figure S1A) was delivered to the Gene Targeted Mouse Service at the University of Cincinnati for creation of the mouse model. The cassette was positioned within *Creld1* to eliminate 1.9 kilobase (kb) of the 5′ untranslated region (UTR), all of exon 1 and a portion of intron 1. Targeted clones of D15T44 ES cell line (from 129/SvEVTac mice from Taconic Labs) were injected into C57Bl blastocytes to create six chimeric male mice. These chimeric mice were bred to NIH Bl/Sw females to generate *Creld1*-heterozygote (*Creld1*^+/−^) mice. Successful elimination of *Creld1* transcription was confirmed by northern blot analysis (Figure S1B). The neighboring upstream and downstream genes were not affected. *Creld1*^+/−^ littermate crosses were done to maintain the *Creld1*-knockout (*Creld1*^−/−^) colony for over 20 generations on the mixed 129-C57 background.

The *Creld1*^−/−^ mouse strain was created and used under approved IACUC protocols. We comply with all institutional and federal policies for the ethical use and treatment of animals and strictly adhere to the Animal Welfare Act, “Guide for the Care and Use of Laboratory Animals” and all USDA and NIH regulations and standards. Compliance was monitored by members of the Department of Comparative Medicine, which is fully accredited by the Association for Assessment and Accreditation of Laboratory Animal Care International.

### 2.4. Mouse Genotyping

Genomic DNA was prepared from tail clips or from yolk sacs using the QIAGEN DNAeasy kit (QIAGEN; Valencia, CA, USA). To detect a *Creld1*-null allele, “KO-F” primer was paired with “Reverse” primer to amplify a 700 base pair fragment. To detect a *Creld1*-wildtype allele, “WT-F” primer was paired with “Reverse” primer to amplify a 528 base pair fragment (Figure S1C). KO-F primer: CCAGTCAAAAACCACAGAGAGGG, WT-F primer: CATCCTTCTCCCCGAGCTGAG, Reverse primer: GTGTTTCCACCCCCGAAGT. PCR reaction: 5 μl SuperMixII (Invitrogen; Carlsbad, CA, USA) + 1 μl genomic DNA + 0.5 μl of each 10 μM primer (KO-F, WT-F and Reverse primer) + 2.5 μl water. Cycling Parameters: 94 °C × 2 minutes + [94 °C × 30 seconds, 60 °C × 30 seconds, 68 °C × 1 minute] × 35 cycles + 68 °C × 3 minutes.

### 2.5. Staging and collecting of embryos

Embryonic day 0.5 (E0.5) was determined by vaginal plug. Pregnant dams were sacrificed for collection of placentas and embryos. Embryonic stage was confirmed by somite count (± 3 somites). Pictures of fresh tissues were taken with a Leica MZ12 dissecting microscope, a Q Imaging MicroPublisher 3.3RTV camera and Rincon Version 7.4 imaging software.

### 2.6. Histological sectioning of mouse tissues

After harvesting, embryos were fixed in 4% formaldehyde (Fisher Scientific F79; Pittsburgh, PA, USA), embedded in Paraplast Plus (McCormick Scientific, LLC; Maryland Heights, MO, USA) and sectioned into 8 μm sections using standard paraffin embedding and sectioning techniques.

### 2.7. Hematoxylin and Eosin (H&E) staining

Sectioned tissues were deparaffinized and Hematoxylin/Eosin (Sigma GHS116, Sigma-Aldrich Corp.; St. Louis, MO/Fischer Protocol 23-314-630, Fisher Scientific; Pittsburgh, PA, USA) staining was performed according to standard procedures in triplicate for each genotype. Tissues were visualized on a Zeiss Axiophot microscope using an Evolution MP COLOR camera by Media Cybernectics and Image Pro Plus 6.3 imaging software.

### 2.8. Alcian Blue and Nuclear Fast Red staining

Sectioned embryos were deparaffinized and Alcian Blue/Nuclear Fast Red staining was performed according to standard procedures in triplicate for each genotype. To quantitate the number of mesenchymal cells in the cushions, three serial sections of two different embryos, matched for developmental stage by somite number (± 3), were counted. Microscopy was performed as described above.

### 2.9. Whole-mount immunohistochemistry

After extraction, placentas and embryos were immediately fixed overnight at 4 °C in 4% formaldehyde (Fisher Scientific F79; Pittsburgh, PA, USA). After fixing, tissues were bleached overnight with 5% H_2_O_2_ (Fisher Scientific H325; Pittsburgh, PA, USA), washed with PBS + 1% Triton X-100 (PBS+1%T) for 5 minutes and incubated twice in PBS + 3% nonfat milk (PBSM) + 1% Triton X-100 (1%T) for an hour at 25 °C. Tissues were then incubated overnight in rat anti-PECAM-1 (Santa Cruz Biotechnology sc-101454; Santa Cruz, CA, USA) in PBSM + 0.1%T at 4 °C. After the incubations, tissues were washed 5 times for 5 minutes in PBSM + 0.1%T before incubating overnight in 1:100 anti-rat IgG HRP (R&D Systems HAF005; Minneapolis, MN, USA) in PBSM + 0.1%T at 4 °C. The tissues were then washed 6 times for 5 minutes in PBSM + 0.1%T then washed with PBS + 0.1%T for 20 minutes. HRP was reacted with DAB (Pierce 1855910 & 1856090, Thermo Scientific; Rockford, IL, USA) washed 3 times with PBS + 0.1%T + 0.2% bovine serum albumin for 5 minutes each, post-fixed with 4% formaldehyde and stored in 70% glycerol at 4 °C. Tissues were imaged with a Leica MZ12 dissecting microscope, a Q Imaging MicroPublisher 3.3RTV camera and Rincon Version 7.4 imaging software. PECAM-IHCs were performed in triplicate for each *Creld1*-genotype and developmental time point.

### 2.10. TUNEL staining

DeadEnd™ Colorimetric TUNEL System (Promega, G7360; Madison, WI, USA) was used to visualize apoptotic cells. To quantitate the number of TUNEL-positive cells, black cells were counted in three serial sections of in the AV-canal and glossopharyngeal arches of representative embryos. Sections were visualized on a Zeiss Axioskop2 motplus microscope with an AxioCam HRM camera and AxioVision 4.6.3 software. TUNEL was performed in triplicate for each *Creld1*-genotype.

### 2.11. Endocardial cushion explant assay

Atrioventricular canals from embryonic day 9.5 mouse hearts were isolated with the aid of a dissecting microscope, cut in half longitudinally and placed on a pre-equilibrated 3-D collagen-matrix and cultured as previously described [11]. The endocardial cushions were allowed to adhere to the matrix overnight. The next day fresh growth media was added with or without supplemental purified mouse-VEGFA 120 protein (R&D Systems AF4116; Minneapolis, MN, USA). Successful mesenchymal cell migration across and into the collagen gels was then assessed by two blinded independent observers, whose observation were then averaged. Endocardial cells were classified upon their appearance as rounded polygonal cells on the collagen-matrix surface with intact cell-to-cell junctions. The invasive mesenchymal cells were characterized by the appearance of stellate-shaped cells within or on top of the matrix. Mandatory recounts were enacted if the independent counts varied by more than 10%. Statistical comparisons between groups are done using Student’s t-test.

### 2.12. VEGFA qPCR

Pooled RNA collected from gender balanced E9.5 mouse heart tubes was reverse transcribed into cDNA using SuperScript III First-Strand Synthesis System (Invitrogen; Carlsbad, CA, USA). Quantitative PCR was performed using the following PCR primers and SYBR green for detection: *VEGFA*, F:CTGGCCAGGCTCCCGATT, R:GATGCCGGTTCCAACCAGAAGTT. *GAPDH* Primers, F:AAATATGACAACTCACTCAAGATTGTC R:CCCTTCCACAATGCCAAAGT. Cycling Parameters: 94 °C × 2 minutes [94 °C × 30 seconds, 60 °C × 30 seconds, 68 °C × 30 seconds] × 50 cycles + 68 °C × 3 minutes.

### 2.13. ELISA assay

ELISA assays were performed using the VEGFA ELISA Kit, Mouse QIA52 (Calbiochem; Darmstadt, Germany) on media from explanted heart after 96 hours in culture, per manufacturer’s instructions. ELISA results were quantified to a standard curve made of the provided purified VEGFA.

## 3. Results

### 3.1. Co-segregation of CRELD1 mutations and VEGFA c.–634allele allele in AVSD

**Figure 1:**
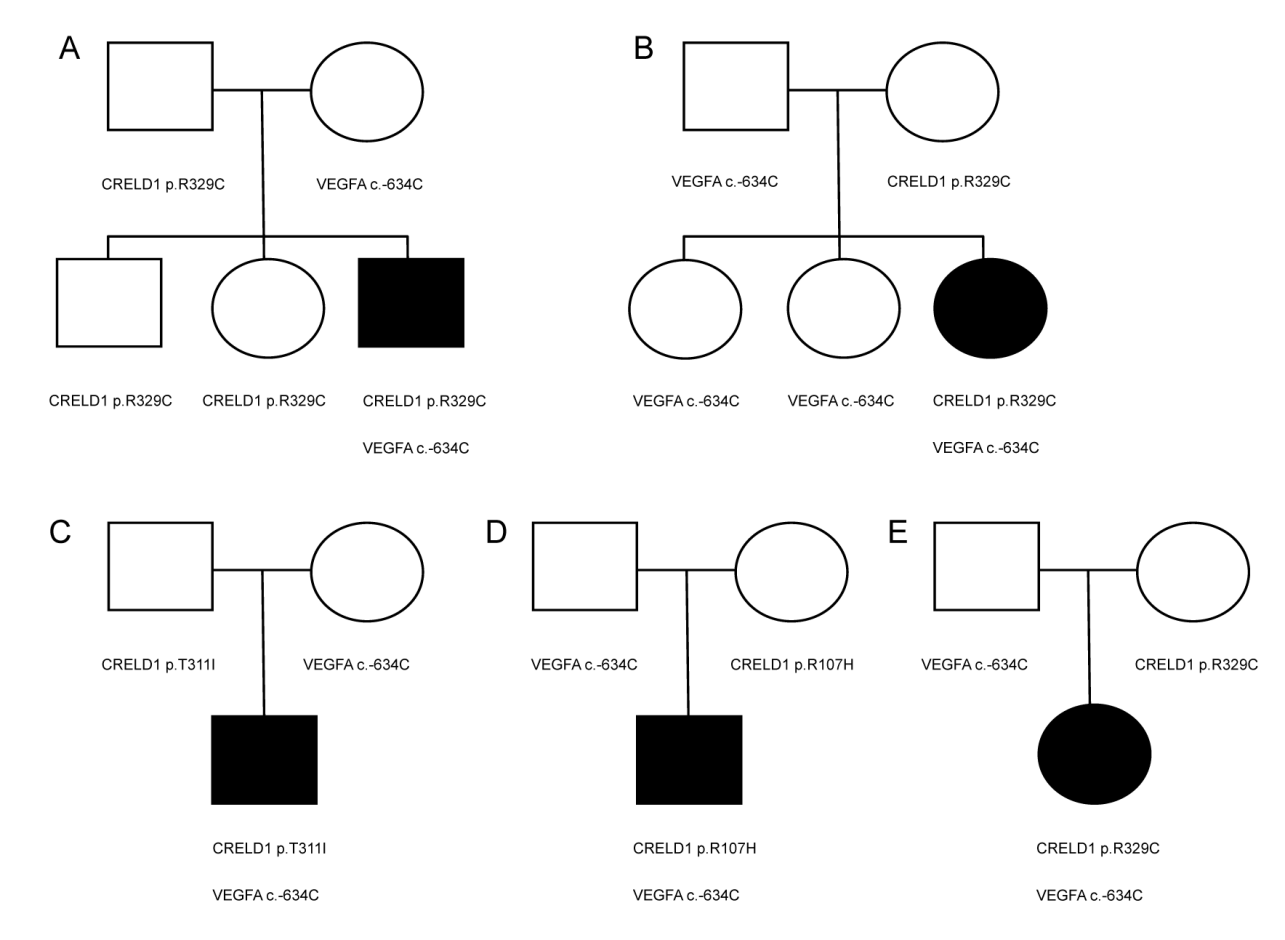
Pedigrees of AVSD families showing co-segregation of *CRELD1* missense mutations and the *VEGFA* c.–634C allele in affected individuals. The AVSD-associated *CRELD1* missense mutations and VEGFA c.–634C genotypes are indicated under each individual’s symbol. All individuals with the *VEGFA* c.–634C allele are heterozygous c.–634GC. All individuals that carry a *CRELD1* missense mutation are homozygous *VEGFA* c.–634GG. Black symbols indicated presence of an AVSD. Note that unaffected family members carry either the *CRELD1*-missense mutation or the *VEGFA* c.–634C allele, but never both. The status of unaffected family members was confirmed by echocardiography for Family A, which has multiple unaffected family members carrying the *CRELD1* missense mutation.

Genotyping of 46 AVSD cases and 50 healthy controls demonstrated a significant association between simplex AVSD and the *VEGFA* c.–634C allele (p-value = 0.02 for trend test based on Table 1). Of the 46 cases 44 had previously been resequenced for *CRELD1*, with heterozygous missense mutations identified in three of those individuals [7], and two additional *CRELD1* mutation positive subjects identified at a later date (Maslen, unpublished data). All AVSD cases with a *CRELD1*-missense mutation also carried the *VEGFA* c.–634C allele (Figure 1). Available family members of the five probands were genotyped for the *CRELD1* missense mutation carried by the proband and the *VEGFA* c.–634 locus. For each family, the proband was the only affected family member. Unaffected parents of AVSD cases carried either the *CRELD1*-missense mutation identified in the proband or the *VEGFA* c.–634C allele, never both. The probands all inherited the *CRELD1* missense mutation from one parent and the *VEGFA* c.–634C allele from the other. Unaffected carriers (parents and siblings) of a *CRELD1*-missense mutation were always homozygous *VEGFA* c.–634GG. In order to assess the statistical significance of this observation, we performed the following calculation. Given 5 probands with both mutations, the probability that all parents carry only one mutation is (0.5)^5^ and the probability that all unaffected siblings carry at most one mutation is (0.75)^4^, for a combined p-value of 0.01.

### 3.2. Knockout of Creld1 was lethal

Complete knockout of *Creld1* resulted in embryonic lethality (Table 2). To determine when *Creld1*^−/−^ death occurred we assessed *Creld1*^−/−^ embryos (Table 3). At E12.5 we found little *Creld1*^−/−^ embryonic tissue, suggesting earlier death. A day earlier at E11.5, we found *Creld1*^−/−^ tissue in the expected Mendelian ratio but embryos were dead, while E10.5 and E9.5 *Creld1*^−/−^ embryos were alive and in the expected Mendelian ratios. Therefore, it appears lethality occurs between E10.5 and E11.5.

### 3.3. Creld1 heterozygotes were normal

*Creld1*^+/−^ pups were born in the expected Mendelian ratio. Mice heterozygous for *Creld1* were grossly normal without anatomical defect, reproduced well, and had life spans comparable to wildtype littermates. Histological analyses of 45 P0 *Creld1*^+/−^ pups and 13 adult *Creld1*^+/−^ hearts found no incidence of cardiac septal defects.

### 3.4. Knockout of Creld1 caused endocardial cushion defects

**Figure 2:**
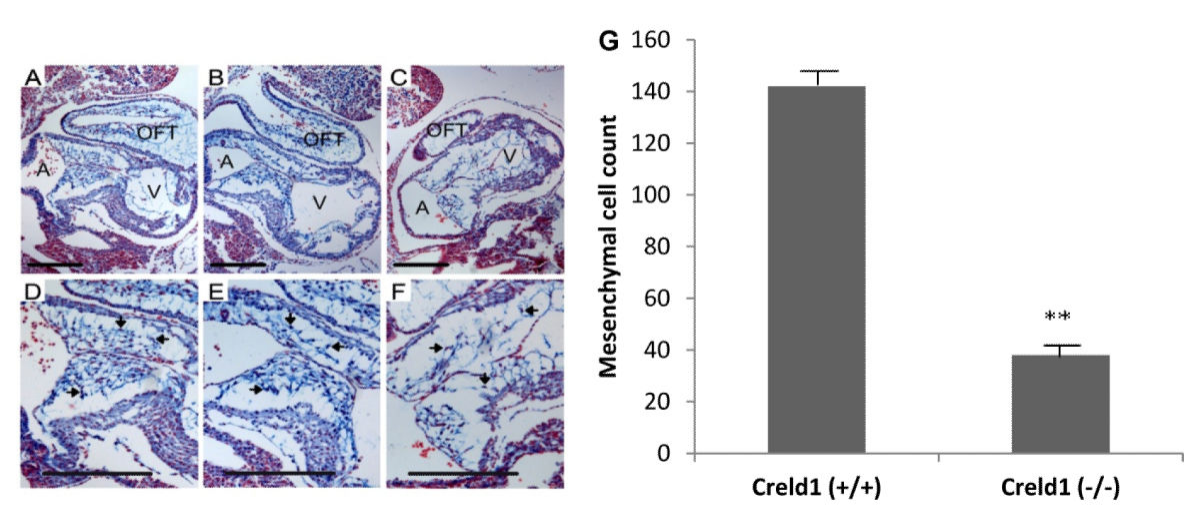
CRELD1 is required for proper AV cushion development. E10.5 mouse hearts stained with Alcian Blue to visualize the extracellular matrix and Nuclear Fast Red to visualize the cells. (A) *Creld1*^+/+^ atrioventricular canal. (B) *Creld1*^+/−^ atrioventricular canal. (C) *Creldl*^−/−^ atrioventricular canal. (D, E. F) enlarged images of the endocardial cushions from panels (A, B, C) respectively. Representative endocardial cushions mesenchymal cells are denoted by arrows. Outflow tract, OFT; common ventricle, V; common atrium, A. Scale bar represents 200 μm. (G) Mean and SEM of the number of mesenchymal cells found in the AV-endocardial cushions. **p value ≥ 0.001.

At E9.5 the *Creldl*^−/−^ hearts were grossly indistinguishable from wild-type littermate hearts (Figure S2) However, at E10.5 *Creldl*^−/−^ hearts showed signs of abnormal development. Histological examination found that *Creldl*^−/−^ atrioventricular endocardial cushions had quantitatively fewer cells and qualitatively less extracellular matrix compared to developmentally matched Creldl^+/−^ and Creldl^+/+^ hearts (Figure 2).

### 3.5. Increased apoptosis in Creldl^−/−^ embryos

**Figure 3:**
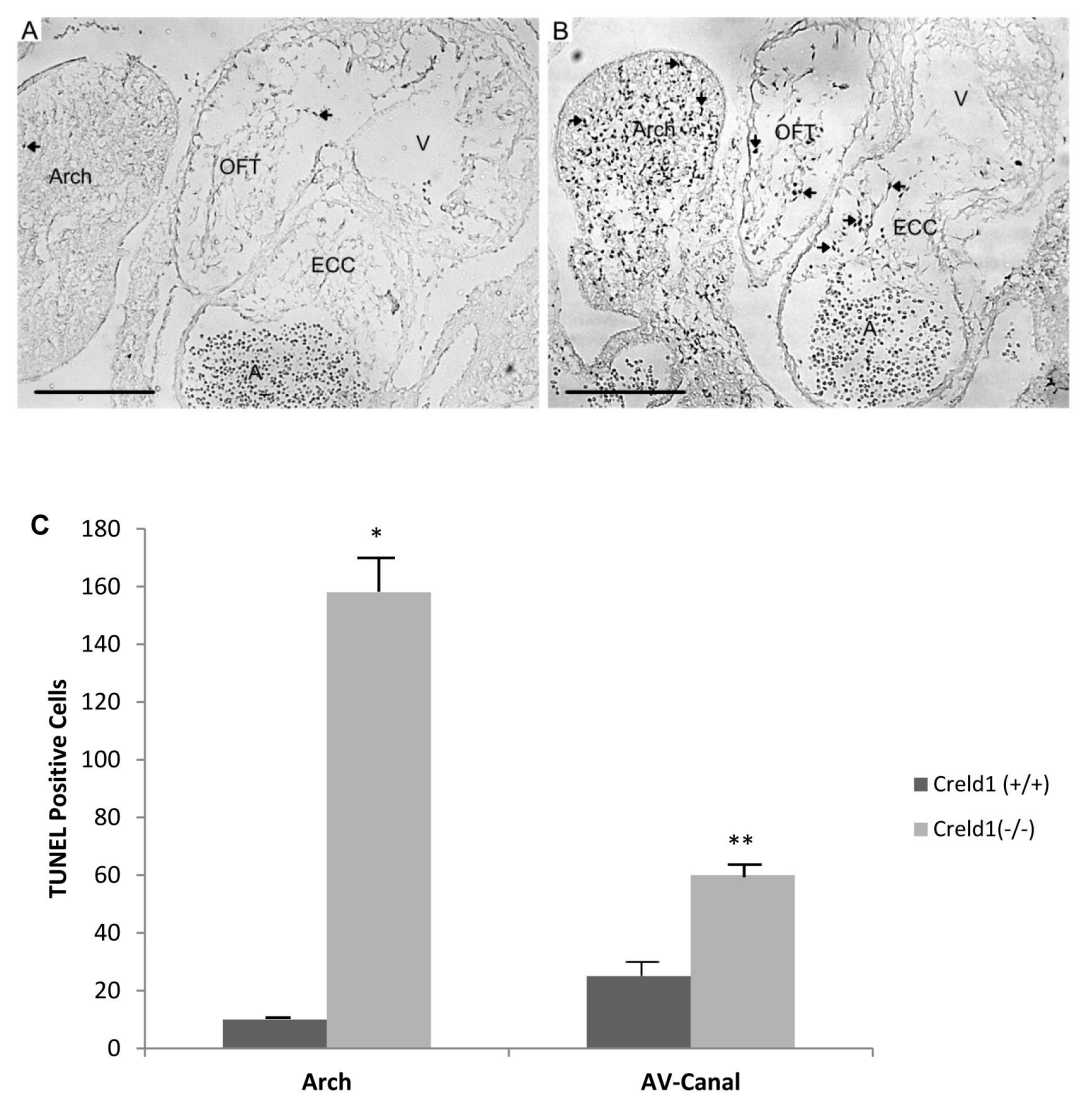
Absence of CRELD1 induces apoptosis. TUNEL staining reveals increased levels of apoptosis in *Creldl*^−/−^ E10.5 embryos. (A) Heart and glossopharyngeal arch of a *Creld1*^+/+^ embryo stained for TUNEL. (B) Heart and glossopharyngeal arch of a *Creldl*^−/−^ embryo stained for TUNEL. Glossopharyngeal arch, Arch; outflow tract, OFT; atrioventricular endocardial cushions, ECC; common atrium, A; common ventricle, V. TUNEL positive cells are black, representative cells are indicated by arrows. Scale bar represents 200 μm. (C) Mean and SEM of the number of TUNEL-positive cells observed in the glossopharyngeal arches and AV-canals. *p value ≤ 0.01, **p value ≤ 0.05.

Cell proliferation appeared normal in E10.5 *Creldl*^−/−^ when compared to wildtype littermates. However, TUNEL staining was increased in E10.5 *Creldf*^−/−^ embryos (Figure 3), most notably in the endocardial cushions and pharyngeal arches. *Creldl*^+/−^ embryos were indistinguishable from wildtype littermates.

### 3.6. Vascular and Structural Abnormalities in *Creldl^(−/−)^* embryos

**Figure 4:**
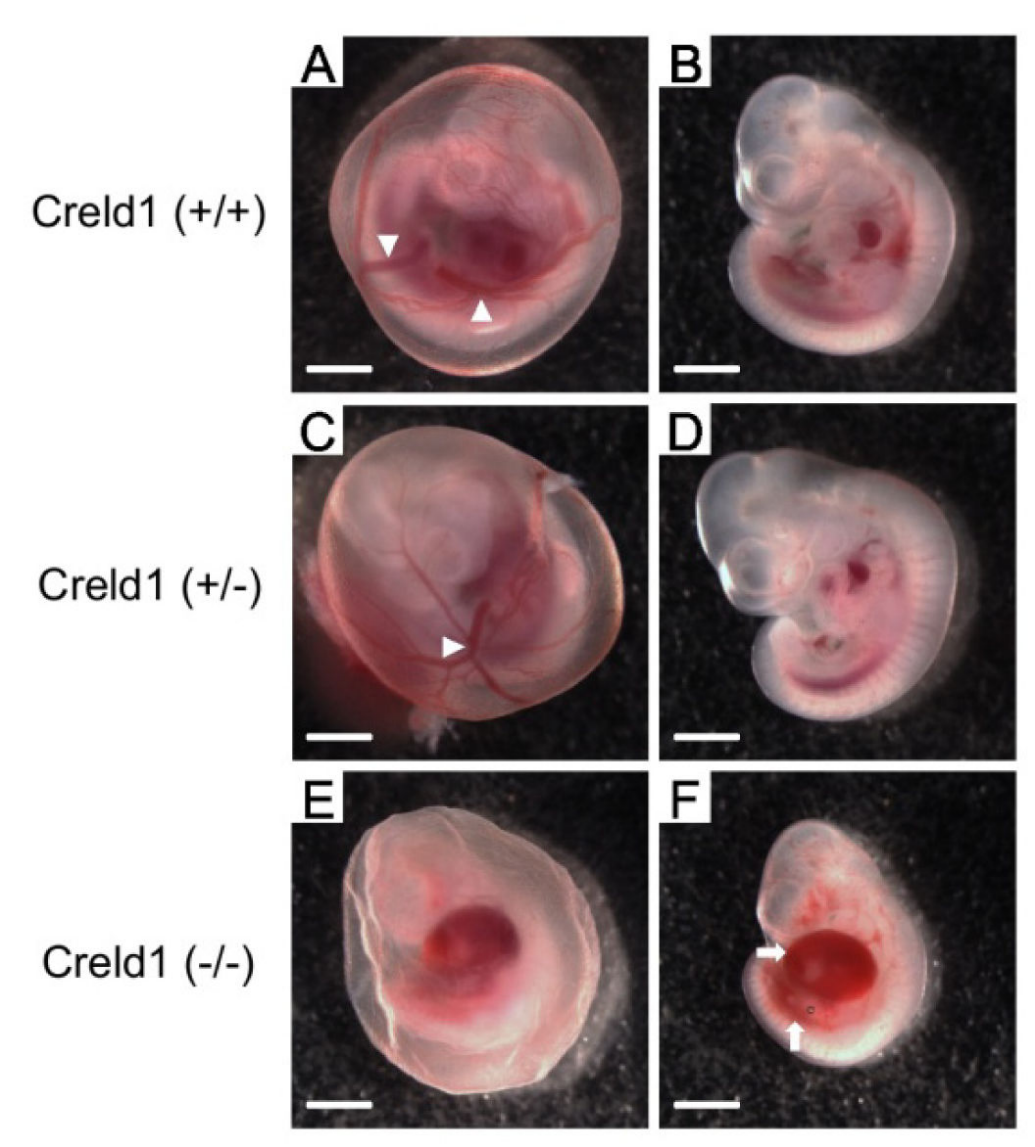
CRELD1 is essential for yolk sac vascularization and embryo development. Representative images of newly harvested E10.5 embryos are shown. (A, C, E) Embryos in yolk sacs. White arrowheads indicate the well developed conducting arteries in the *Creld1*^+/+^ and *Creld1*^+/−^ yolk sacs (A, C). (E) *Creld1*^−/−^ yolk sac, showing the absence of large vessels. (B, D, F) Embryos without yolk sacs. Note the hemorrhaging and enlargement of pericardial sac in the *Creld1*^−/−^ embryo, as marked by white arrows (F). Scale bars represents 1mm.

**Figure 5:**
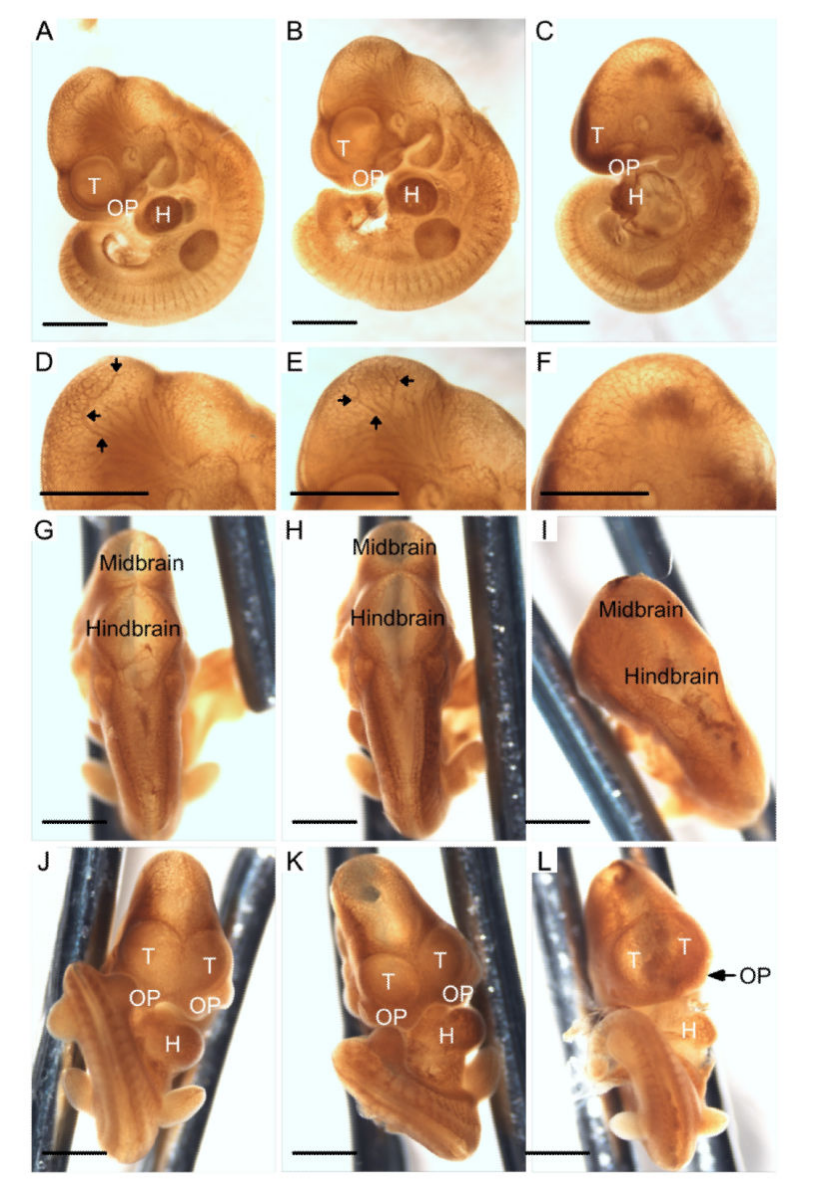
CRELD1 is required for proper embryonic development. Whole E10.5 embryos visualized by PECAM-staining reveal defects in embryonic development. (A, D, G, J) Images of a representative *Creld1*^+/+^ embryo showing stage-appropriate development. (A) A side view of the whole embryo. (D) A close-up view of the cranial region. (G) A dorsal view showing the midbrain and hindbrain. (J) Front view showing craniofacial structures and the heart. (B, E, H, K) Images of a *Creld1*^+/−^ embryo, (B) side view, (E) close-up of the cranial region, (H) dorsal view showing the midbrain and hindbrain, (K) front view showing craniofacial structures and the heart. Black arrows indicate the well developed cephalic venous plexus of the *Creld1*^+/+^ and *Creld1*^+/−^ embryos (D, E). (C, F, I, L) Representative images of a *Creld1*^−/−^ embryo. (C) Note that the embryo is smaller than the *Creld1*^+/+^ and *Creld1*^+/−^ littermates (A, B). Poor development of the craniofacial region is also evident. (F) The *Creld1*^−/−^ vascular plexus remained more primitive, with no evidence of arborization of the vasculature and a lack of a well-developed forebrain. (I) The dorsal view of the *Creld1*^−/−^ embryo shows disorganization of tissue at the midline and under-developed midbrain and hindbrain. (L) *Creld1*^−/−^ embryos had underdeveloped telencephalons and olfactory placodes compared to *Creld1*^+/+^ and *Creld1*^+/−^ embryos (J, K). Olfactory placode, OP; telencephalon, T; heart, H. Scale bars represent 1mm.

*Creldl*^−/−^ yolk sac vascular maturation was impaired (Figure 4). Vascularization was reminiscent of an “orange peel”, a phenotype previously described in the *delta-like 4* (a NOTCH ligand) knockout mice [19]. PECAM immunohistochemistry revealed that while *Creld1*^+/+^ and *Creld1*^+/−^ embryos had fully developed and well-organized cephalic vascular trees at E10.5, *Creld1*^−/−^ cephalic vascular trees failed to fully arborize and appeared more primitive (Figure 5 A-F). PECAM staining also revealed that the *Creld1*^−/−^ forebrain and craniofacial features were severely underdeveloped (Figure 5 G-L) compared to somite matched *Creld1*^+/+^ embryos. The midbrain and hindbrain also had gross structural abnormalities and tissue at the midline appeared disorganized. Overall, these gross abnormalities are found in the regions of highest CRELD1 expression.

### 3.7. Interaction between CRELD1 and VEGFA in endocardial cushion development

Endocardial cushion tissue was explanted and cultured on a collagen matrix to monitor the ability of the tissue to successfully complete an epithelial-to-mesenchymal transformation (EMT) as a proxy for early development in atrioventricular valve morphogenesis. Specifically this system permits quantification of the endocardial cushion’s ability to undergo this critical step in early valve and septa formation under varied conditions. Successfully transformed mesenchymal cells can be tallied in this assay as they migrate away from the explanted tissue and invade collagen matrix.

**Figure 6:**
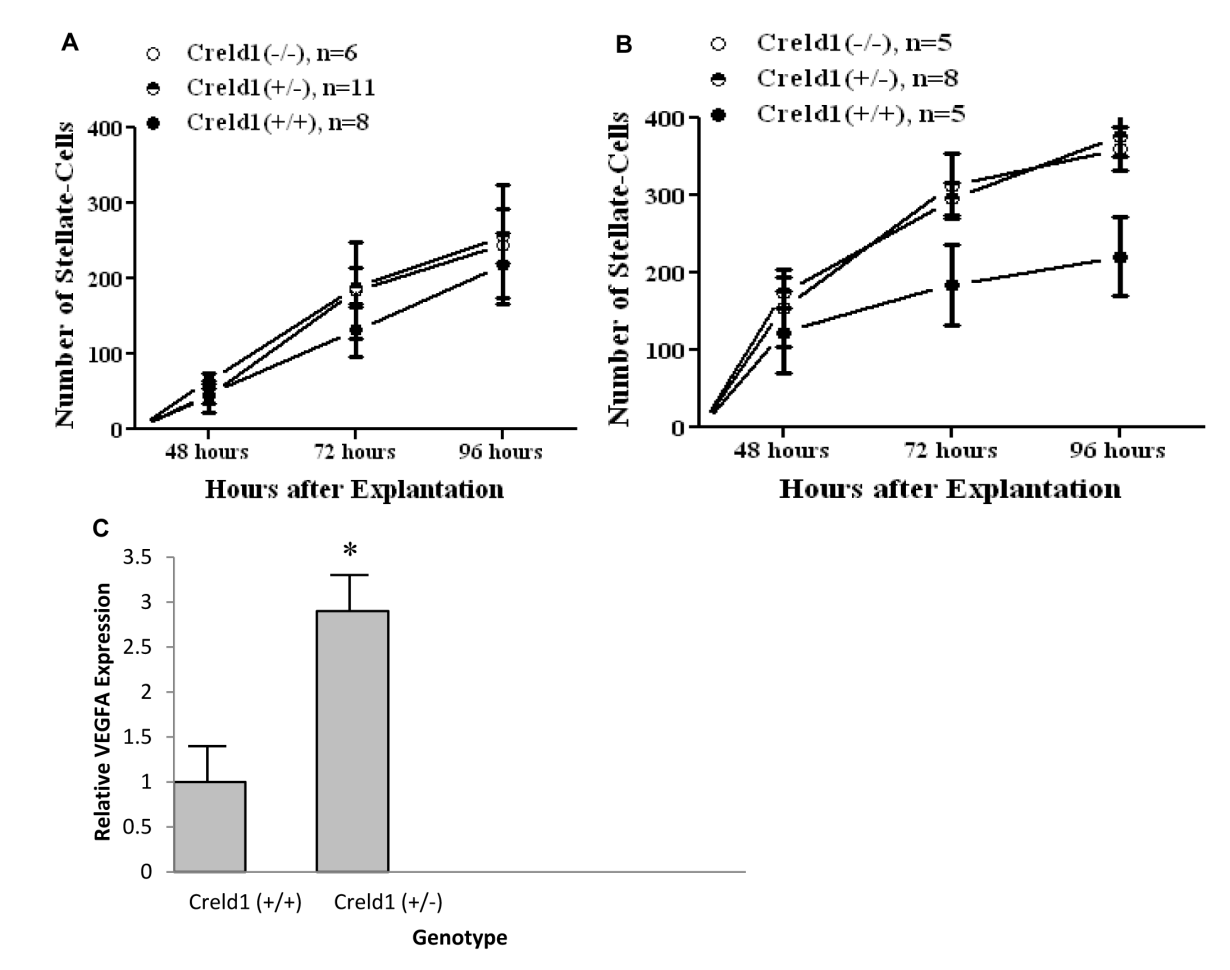
CRELD1 controls cell response to VEGFA during AV-canal morphogenesis. Graphical representation of results from endocardial cushion morphogenesis assays. Explanted AV cushions successfully undergo EMT in culture, with migration of mesenchymal cells (stellate shaped cells) into the surrounding matrix as a measure of EMT. (A) Under endogenous levels of VEGFA, the stellate cell counts from the explanted *Creldl*^−/−^, *Creld1*^+/−^ and *Creld1*^+/+^ atrioventricular (AV) canals were indistinguishable at all time points. (B) Addition of *VEGFA* caused the *Creld1*^−/−^ and *Creld1*^+/−^ explants to have abnormally high levels of mesenchymal cell migration compared to the *Creld1*^+/+^ explants at both 72 hours (*p* value ≤ 0.1) and at 96 hours (*p* value ≤ 0.05). (C) Quantitative analysis of *VEGFA* expression in embryonic hearts. *VEGFA*-RNA was 3-fold higher in E9.5 *Creld1*-deficient *Creld1*^−/−^ mouse hearts compared to wildtype. *p value ≤ 0.01.

Under unmodified growth conditions (media containing < 15 ng/ml of VEGFA) the *Creldl* genotype had no effect on the number of mesenchymal cells observed migrating from the explanted tissue (Figure 6A). However, Creld1-deficient endocardial cushion explants grown under higher VEGFA levels (200 pg/ml), mimicking the VEGFA overexpression associated with the *VEGFA*-634C allele, reacted abnormally. The counts for migrating mesenchymal cells from *Creld1*^+/−^ and *Creldl*^−/−^ endocardial cushions were significantly higher than those from wildtype littermates. The wildtype explants were unaffected by the addition of VEGFA (Figure 6B). Additionally, we found that E9.5 *Creld1*^+/−^ hearts had abnormality high *VEGFA* expression, nearly 3 times higher than that found for *Creld1*^+/+^ littermates (Figure 6C).

## 4. Discussion

Congenital heart defects such as AVSD can occur as a phenotypic component of a syndrome, but also as non-syndromic events. These are often simplex cases, although the frequency of affected related individuals spread across generations suggests that heritability is high [20]. Rare missense variants, including mutations in *CRELD1*, have been associated with AVSD in both syndromic and non-syndromic cases [5,6,7,21]. Incomplete penetrance has been demonstrated for *CRELD1* mutations, which is consistent with our proposed disease threshold model for AVSD [5,8]. In this model genetic, epigenetic, and/or environmental risk factors must co-occur in unknown combinations to breach the threshold for disease for AVSD to manifest. Importantly, we have shown that inactivating mutations in *Creld1* confer significant risk for the development of cardiac septal defects when crossed with the highly susceptible TS65Dn mouse model for Down syndrome [8]. This confirmed that genetic risk factors such as trisomy for genes on human chromosome 21 and *CRELD1* mutations can act in an additive or synergistic fashion to modify the risk of CHD. However, in the absence of a single modifier of very large effect like trisomy 21, additional risk factors may be necessary to cause heart defects.

There is growing evidence that rare variants play a significant role in the cause of AVSD. In particular, we recently demonstrated that there is a concentration of AVSD-associated rare variants in VEGFA pathway genes suggesting that this may be a predominant pathway in the cause of AVSD [5]. However, the potential for contribution from common variants also exists. In this study we hypothesized that there was an interaction between inactivating mutations in *CRELD1* and a common *VEGFA* functional SNP based on a significant allelic association between the two genes in individuals with AVSD. VEGFA is known to be an important morphogen in the control of early heart valve development [11,12,22], but there was no established function for CRELD1 in heart development other than evidence of expression during embryogenesis [23]. Consequently, we used a *Creld1* knockout mouse model to characterize the role of CRELD1 in cardiovascular development and to test for interaction between increased VEGFA expression and deficiency of CRELD1.

Basic characterization of Creld1-null embryos indicates that CRELD1 plays a substantial role in cardiovascular development. Of particular interest was the hypocellularization of the atrioventricular (AV) endocardial cushions and substantial increase in apoptosis in the cushions and elsewhere. One of the hallmark features of AV valve development is the process by which the endocardial cushions of the atrioventricular canal begin morphing into the valves by undergoing an epithelial to mesenchymal transformation. This developmental event has been studied extensively using an explant assay to follow valve morphogenesis in vitro [11,22,24,25]. Using this assay, we demonstrated an interaction between VEGFA and CRELD1 during atrioventricular canal morphogenesis, where there was a significant increase in mesenchymal cell invasion into the matrix surrounding the explanted endocardial cushions from mice with reduced CRELD1 expression. This suggested that the programmed change in cell proliferation and migration following the epithelial to mesenchymal transformation is altered when there is a deficiency of CRELD1 coupled with an increase in VEGFA. The increase in mesenchymal cell infiltration of surrounding matrix indicates that the cell response to VEGFA is controlled by CRELD1. These results provided biological context for the allelic association between inactivating mutations in *CRELD1* and the functional *VEGFA* c.–634C allele in individuals with AVSD. Although the focus here is on endocardial cushion development, there is substantial evidence that earlier developmental events in the second heart field control atrioventricular septation. A role for CRELD1 in that process is currently unknown, but cannot be ruled out. It is also possible that there is a combined contribution of defective endocardial cushion development and defective development of the DMP resulting in AVSD. Further study of CRELD1 in early heart development is warranted.

Like the incomplete penetrance seen for heterozygous *CRELD1* missense mutations in humans, the haploinsufficient *Creld1*^+/−^ mouse is phenotypically normal, but displays biochemical abnormalities that predispose the developing heart to an aberrant response to increased VEGFA signaling. Additional characterization of the Creld1 mouse model gives further insight into the biological processes controlled by CRELD1, including a significant increase in VEGFA expression in CRELD1-deficient embryonic hearts. Abnormal vasculogenesis in the *Creld1*^−/−^ embryos is further suggestive of interaction with VEGFA. Consequently, it appears that CRELD1 plays a role in regulating VEGFA and that CRELD1 haploinsufficiency alone causes dysregulation of VEGFA. This coupled with constitutively increased VEGFA expression from the *VEGFA* c.–634C allele may tip the balance in heart development towards a cascade of events that result in a malformed heart. Whether or not the *CRELD1-VEGFA* allelic interaction is sufficient to be fully penetrant remains an open question, but it appears that it has the potential to be a contributing factor in the pathogenesis of AVSD. The data presented for the families with an AVSD proband suggests, but does not prove, a causative relationship between the *CRELD1* mutation and *VEGFA* functional SNP. Given the small number of families with *CRELD1* mutations available for study, it is possible that the allelic association is spurious and care should be taken in interpreting this type of human genetics data. Further studies are needed to determine the significance of this finding. Identification of pathogenic interactions between rare variants and a functional SNP using a combination of human genetics and mouse models is a necessary step in significantly advancing our understanding of the genetic events that lead to CHD in humans.

## Supplementary Material

Supplemental Data

## Figures and Tables

**Table 1:** *VEGFA* c.–634C allele is over-represented in AVSD cases compared to controls.

VEGFA Genotype	Controls	Cases
c.-634GG	30	18
c.-634GC	17	21
c.-634CC	3	7

The *VEGFA* c.–634C allele was enriched in the sporadic AVSD population compared to the Coriell Caucasian Human Variation Control population (*p value* = 0.02, based on Armitage’s trend test).

**Table 2:** Pup survival.

*Creld1* Genotype	+/+	+/+	+/−	+/−	−/−	−/−
Gender	Male	Female	Male	Female	Male	Female
Total number born	61	57	106	121	0	0
Percent by genotype and gender	18%	17%	31%	35%	0%	0%
Percent of total pups born	34%		66%		0%	

Pup survival was tallied 3 weeks post birth. 67 litters with an average of 4.64 pups per litter were assessed. Complete knockout of Creld1 was embryonic lethal with no live births of Creld1−/− pups. Wildtype and Creld1+/− mice were born in expected Mendelian ratios. There was no significant difference in gender ratios for any genotype.

**Table 3:** Frequency of Creld1-genotypes during embryogenesis.

	*Creld1* Genotypes		
Embryonic Day	−/−	+/−	+/+
**9.5**	51 (20.4%)	125 (50%)	74 (29.6%)
**10.5**	92 (22.2%)	214 (51.6%)	109 (26.3%)
**11.5**	12 (24%)^a^	28 (56%)	10 (20%)
**12.5**	3 (15%)^b^	11 (55%)	6 (30%)

a The E11.5 *Creld1*^−/−^ embryos did not have beating hearts.

b The E12.5 *Creld1*−/− tissue found at this stage was being reabsorbed.

Incidence of Creld1genotypes during mouse embryogenesis. E10.5 and E9.5 Creld1^−/−^ embryos were alive and in Mendelian ratio. E11.5 Creld1^−/−^ embryos were in concordance with Mendelian ratio but dead. By E12.5 little Creld1^−/−^ tissue was identifiable and by E13.5 no Creld1^−/−^ embryonic tissue was found. Ratio conformance tested by chi-square analyses (E9.5, χ^2^ = 4.232, p-value = 0.1205; E10.5 embryos, χ^2^ = 1.800, *p*-value = 0.4066; E11.5 embryos, χ^2^ = 0.880, *p*-value = 0.6440).
