# Supplementary material for: Allelic Interaction between CRELD1 and VEGFA in the Pathogenesis of Cardiac Atrioventricular Septal Defects
Source: AIMS Genet. Author manuscript; Available in PMC 2014 Oct 17. (PMC4200510; doi:10.3934/genet.2014.1.1#sthash.jksuJTeC.dpuf)
Supplement: Supplemental Data [file NIHMS592208-supplement-Supplemental_Data.pdf]

Supplemental Figures

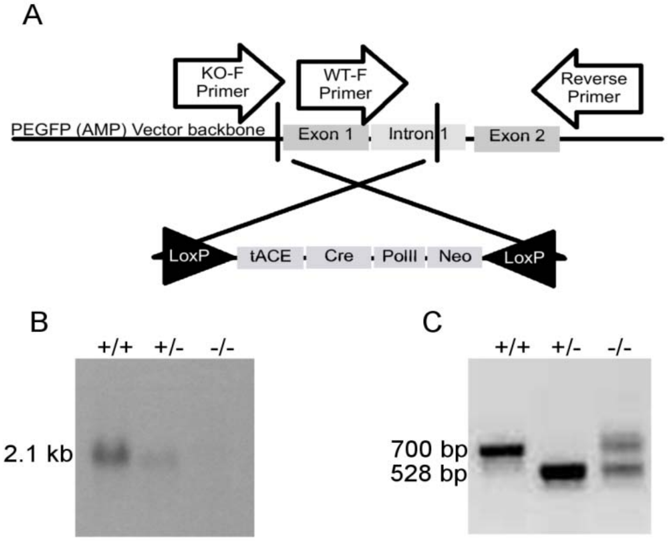

Figure S1: A schematic diagram of the *Creld1* targeting vector. Recombination was driven by the self-excising tACE/Cre/Neo cassette, which disrupted 1.9 kb of the 5' untranslated region (UTR), exon 1 and part of intron1 of the mouse *Creld1* gene. Also illustrated are the locations of the allele-specific primer binding sites that were used for genotyping. Testis-specific angiotensin-converting enzyme promoter, tACE; cre-recombinase, cre; RNA polymerase II, PolII; neomycin, Neo. **B**: Northern blot showing knockdown of *Creld1*-RNA expression in the *Creld1*-knockout mouse colony. **C**: Image of the allele-specific PCR used to genotype the *Creld1*-knockout mouse colony.

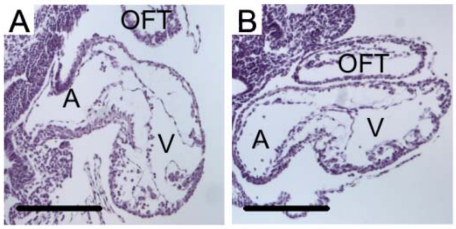

Figure S2: H&E stained sectioned outflow tract and atrioventricular cushions of E9.5 (A) *Creld1*$^{(+/+)}$ and (B) *Creld1*$^{(-/-)}$ embryos. There are no appreciable differences in the heart between the two genotypes at this stage of development. Common ventricle, V; common atrium, A; outflow tract, OFT. Scale bar represents 200μm.

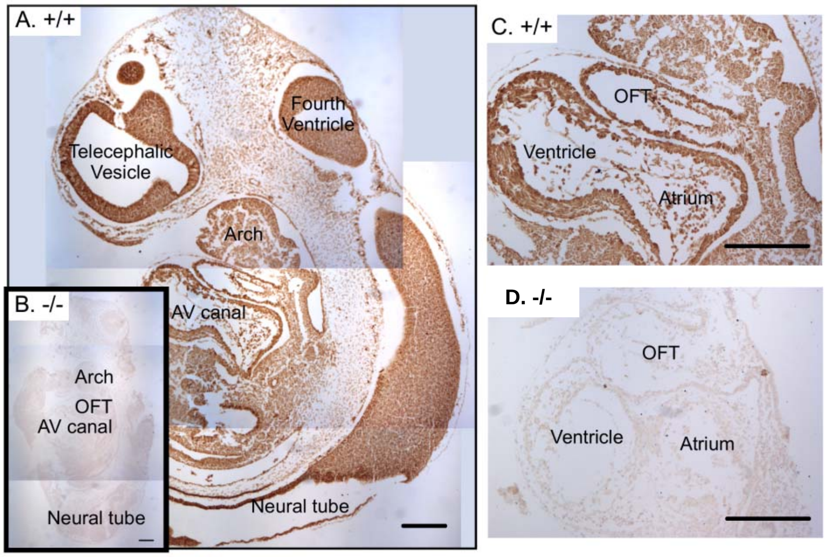

Figure S3. Localization of CRELD1 in E10.0 embryos. Immunohistochemical staining was done using a CRELD1-specific antibody (Santa Cruz Biotechnology) using standard techniques with HRP detection. (A) Image from a *Creld1*$^{+/+}$ embryo showing intense staining in the CNS, heart and arches. (C) Close-up view of the heart. (B,D) *Creld1*$^{-/-}$ embryo sections as negative controls showing lack of CRELD1 staining.
